# Interferons Are Pro-Inflammatory Cytokines in Sheared-Stressed Human Aortic Valve Endothelial Cells

**DOI:** 10.3390/ijms221910605

**Published:** 2021-09-30

**Authors:** Iván Parra-Izquierdo, Tania Sánchez-Bayuela, Javier López, Cristina Gómez, Enrique Pérez-Riesgo, J. Alberto San Román, Mariano Sánchez Crespo, Magdi Yacoub, Adrian H. Chester, Carmen García-Rodríguez

**Affiliations:** 1 Instituto de Biología y Genética Molecular, Spanish National Research Council (CSIC), Universidad de Valladolid, 47003 Valladolid, Spain; ivanparraizquierdo@gmail.com (I.P.-I.); tania.sanchez-bayuela@alumnos.uva.es (T.S.-B.); crisgl@ibgm.uva.es (C.G.); epercamh@gmail.com (E.P.-R.); mscres@ibgm.uva.es (M.S.C.); 2 ICICOR, Hospital Clínico Universitario, 47005 Valladolid, Spain; javihouston@yahoo.es (J.L.); asanroman@secardiologia.es (J.A.S.R.); 3 Centro de Investigación Biomédica en Red en Enfermedades Cardiovasculares (CIBERCV), 28029 Madrid, Spain; 4 National Heart & Lung Institute, Imperial College London, London SW3 6LR, UK; m.yacoub@imperial.ac.uk; 5 Magdi Yacoub Institute, Harefield UB9 6JH, UK

**Keywords:** valvular endothelial cells, interferons, monocyte adhesion, inflammation, JAK/STAT

## Abstract

Calcific aortic valve disease (CAVD) is an athero-inflammatory process. Growing evidence supports the inflammation-driven calcification model, mediated by cytokines such as interferons (IFNs) and tumor necrosis factor (TNF)-α. Our goal was investigating IFNs’ effects in human aortic valve endothelial cells (VEC) and the potential differences between aortic (aVEC) and ventricular (vVEC) side cells. The endothelial phenotype was analyzed by Western blot, qPCR, ELISA, monocyte adhesion, and migration assays. In mixed VEC populations, IFNs promoted the activation of signal transducers and activators of transcription-1 and nuclear factor-κB, and the subsequent up-regulation of pro-inflammatory molecules. Side-specific VEC were activated with IFN-γ and TNF-α in an orbital shaker flow system. TNF-α, but not IFN-γ, induced hypoxia-inducible factor (HIF)-1α stabilization or endothelial nitric oxide synthase downregulation. Additionally, IFN-γ inhibited TNF-α–induced migration of aVEC. Also, IFN-γ triggered cytokine secretion and adhesion molecule expression in aVEC and vVEC. Finally, aVEC were more prone to cytokine-mediated monocyte adhesion under multiaxial flow conditions as compared with uniaxial flow. In conclusion, IFNs promote inflammation and reduce TNF-α–mediated migration in human VEC. Moreover, monocyte adhesion was higher in inflamed aVEC sheared under multiaxial flow, which may be relevant to understanding the initial stages of CAVD.

## 1. Introduction

Calcific aortic valve disease (CAVD) has emerged as a major and increasingly prevalent valvulopathy worldwide. The lack of a pharmacological treatment to halt or reverse its development reinforces the necessity of identifying new and safe druggable targets. Over the last decades, a series of clinical, in vivo, and in vitro studies have pointed to inflammation as a major mechanism of CAVD at its early stages [1,2,3,4,5,6]. While the trigger for the inflammatory response remains elusive, the association of lipoprotein (a) and oxidized LDL with CAVD suggest that they may play a role in the pathogenesis of the disease [7,8]. Levels of oxidized LDL have been correlated with fibrocalcific remodeling, the density of inflammatory cells and expression of tumor necrosis factor (TNF-α) [8]. Furthermore, valve fibrosis has been correlated with the expression of IL-6 in CAVD [9].

In valve endothelial cells (VEC), TNF-α is known to induce inflammation and oxidative stress [10] as well as endothelial to mesenchymal transition, a process also promoted by IL-6 [11,12]. More recently, the immune cytokines type I and II interferons (IFNs) have been shown to promote inflammation and calcification in valvular interstitial cells (VIC) [13,14]. However, the effects of IFNs in VEC remain unknown. Type I and II IFNs, downstream Janus kinases (JAK), and signal transducers, such as activators of transcription (STAT), play a key role in regulating the immune and inflammatory response [15,16]. In the aortic valve, a recent report has shown that IFN-γ is expressed in calcified regions of diseased human valves [17].

Aortic valve endothelium, which is continuously exposed to hemodynamic forces, is essential to maintain valve integrity and homeostasis [18]. Most evidence pointing to a protective role for VEC in CAVD comes from both in vitro and ex vivo models [19,20,21]. However, in early stages of CAVD, an unresolved endothelial damage, characterized by increased oxidative stress as well as immune cell and lipid infiltration may lead to endothelial dysfunction [4]. Remarkably, calcification develops mainly in the fibrosa layer of aortic valves [1,4,22], in a process that may be regulated by specific differences between endothelial cells of the aortic side (aVEC) or the ventricular side (vVEC). Significant hemodynamic differences occur in either side of the aortic valve. The aortic surface is exposed to oscillatory low-shear flow and the ventricular side to laminar, high-shear flow [22], which is thought to promote the presence of athero-resistant regions within the vascular system [23]. Side-specific differences in nitric oxide (NO) signaling, mechanical properties and transcriptional profile have been reported, with aVEC expressing lower levels of protective and anti-calcifying genes [21,24,25]. However, the influence of side-specific and shear stress pattern on the response of VEC to inflammatory cytokines has not yet been investigated.

The aim of this study was to investigate the effects of IFNs in human VEC, focusing on inflammation, immune cell adhesion and cell migration, and comparing the effects with the canonical pro-inflammatory cytokine TNF-α. Additional aims include analyzing potential side-specific differences and the influence of flow patterns. This information could provide new insights into the effects of cytokines and their interactions in VEC, which could be relevant to understanding CAVD initiation.

## 2. Results

### 2.1. IFNs Activate JAK/STAT Signaling in Human VEC under Static Conditions

**Figure 1:**
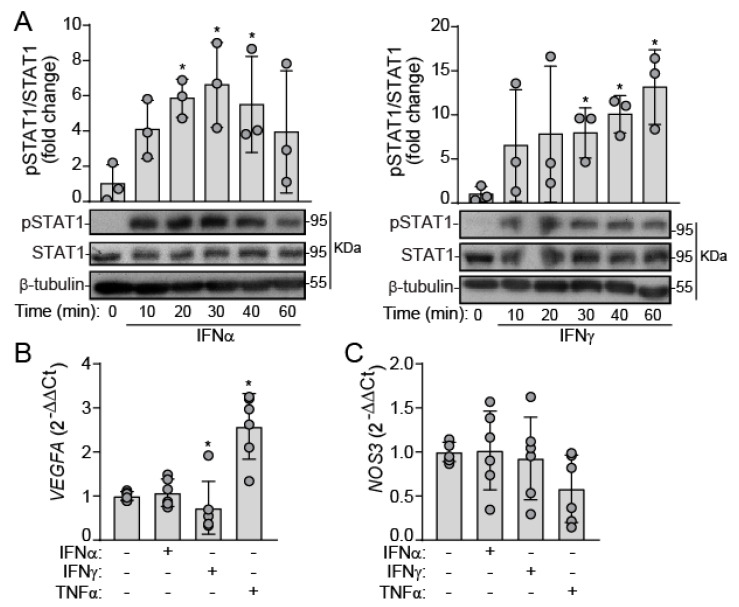
Type I and II interferons promote STAT1 activation but do not affect *VEGF* and *NOS3* expression in VEC. (**A**–**C**) Mixed VEC monolayers were challenged with the indicated ligands under static conditions for the specified times. (**A**) Western blot analysis of STAT1 canonical activation (phosphorylation at tyrosine 701); n = 3 isolates from independent valve donors. To quantitatively compare samples from different blots, Western blot data were expressed as fold change. (**B**,**C**) qPCR analysis of *VEGFA* (**B**) and *NOS3* (**C**) gene expression after 24 h activation; n = 6. IFNα indicates 100 ng/mL IFN-α; IFNγ, 100 ng/mL IFN-γ; TNFα, 5 ng/mL TNF-α. Datasets A–C: Mean ± S.D. One-Way ANOVA statistics, followed by the Dunnett post-hoc test (vs. control), as indicated in Methods. * *p* < 0.05 compared with the control group.

First, we sought to investigate whether human VEC are responsive to type I and II IFNs. We used mixed VEC populations composed of cells from both the aortic and ventricular sides of the valve, which were incubated with recombinant type I and II IFNs (IFN-α and IFN-γ respectively) under static conditions. Western blot analysis revealed that both IFN-α and IFN-γ promoted STAT1 phosphorylation at tyrosine 701 residue in VEC (Figure 1A). To elucidate the IFN-induced phenotype, we analyzed the expression of genes relevant to endothelial function, i.e., vascular endothelial growth factor A (*VEGFA*) and endothelial NO synthase (*NOS3*). We used TNF-α as positive control. qPCR analysis disclosed that IFNs did not affect VEGFA and NOS3 expression. In contrast, TNF-α induced *VEGFA* upregulation (Figure 1B,C), pointing to differential effects between the two cytokines in human VEC.

### 2.2. IFNs Induce NF-κB Activation and Inflammatory Molecule Expression under Static Conditions

**Figure 2:**
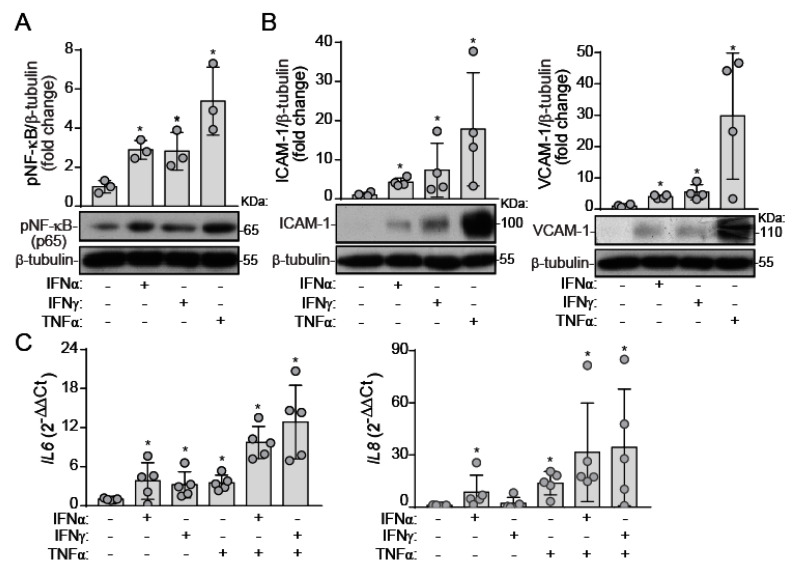
Interferons act as pro-inflammatory cytokines in VEC. (**A**–**C**) VEC monolayers were challenged with the indicated ligands under static conditions for 24 h. (**A**,**B**) Cell lysates were analyzed by Western blot to assess NF-κB activation (**A**) and adhesion molecule expression, ICAM-1 and VCAM-1 (B); n = 4. (**C**) qPCR analysis of *IL6* and *IL8* gene expression; n = 5. Doses as in Figure 1. Datasets A–C: Mean ± S.D. One-Way ANOVA statistics, followed by the Dunnett post-hoc test. * *p* < 0.05 compared to the control group.

Our next step was to investigate the inflammatory potential of IFNs, recently reported as pro-inflammatory cytokines in human VIC [13,14]. In mixed VEC under static conditions, IFN-α and IFN-γ promoted the activation of the master transcription factor for inflammation, NF-κB (Figure 2A). Consistently, IFNs up-regulated the expression of downstream genes, i.e., intercellular cell adhesion molecule (ICAM)-1 and vascular cell adhesion molecule (VCAM)-1 (Figure 2B). TNF-α was the most potent pro-inflammatory cytokine (Figure 2A,B). Moreover, qPCR analysis revealed that IFN-α and IFN-γ increased *IL6* gene expression. However, only IFN-α increased *IL8* expression, pointing to IFN-type specificity in the response of VEC (Figure 2C). Based on the expression of IFN-γ and TNF-α in calcified human valves [17,26], we also explored a potential interplay between IFNs and TNF-α on IL expression, but no cooperation was observed (Figure 2C). Altogether, data unveiled type I and II IFN as pro-inflammatory cytokines in VEC isolated from human non-calcified valves.

### 2.3. TNF-α, but Not IFN-γ, Induces HIF-1α Expression and Downregulation of NOS3 in Side-Specific VEC under Shear Stress

After studying the pro-inflammatory effects of IFNs in mixed VEC populations, we next investigated potential specific responses to IFN-γ in VEC isolated specifically from the aortic or the ventricular side of the valve on the expression of hypoxia-induced factor (HIF)-1α. We focused on IFN-γ, given its association with atherosclerosis and its detection in calcified human valves [17,27]. To simulate different patterns of shear, we cultured and activated the cells in an orbital shaker swirling system that generates a difference in the amount of shear stress between the edge (uniaxial) and the center (multidirectional).

**Figure 3:**
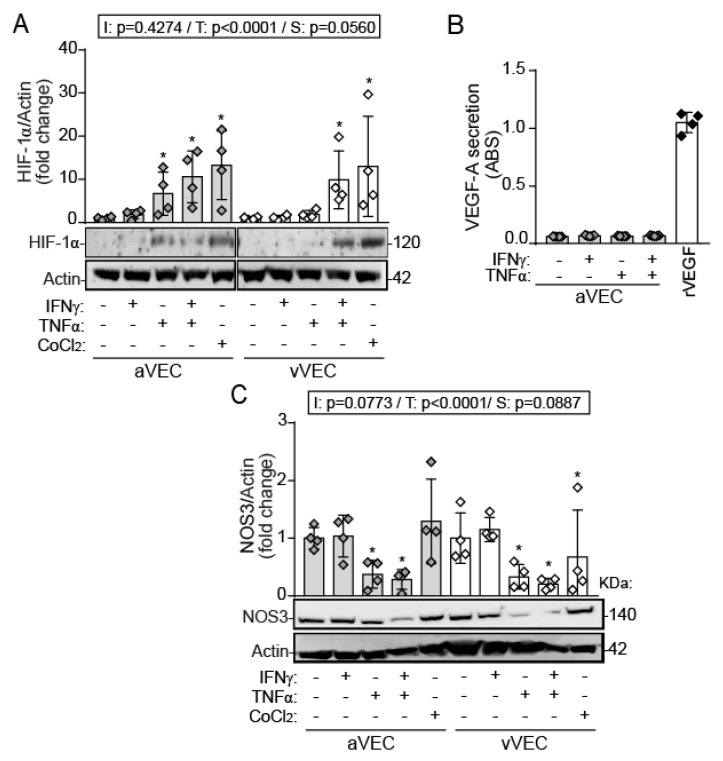
TNF-α but not IFN-γ, promoted the immune induction of HIF-1α, as well as NOS3 protein downregulation in side-specific VEC. (**A**–**C**) aVEC and vVEC were sheared for 48 h and then treated with the indicated ligands for 24 h. (**A**) Whole cell extracts were analyzed by Western blot for HIF-1α expression. Densitometry analysis and representative blot of n = 4 aVEC and vVEC pairs from independent valve donors. Cropped images are from the same membrane and were processed in parallel. (**B**) Supernatants from activated aVEC were analyzed to assess VEGF-A secretion. ABS indicates raw absorbance at 450 nm. (**C**) Whole cell extracts were analyzed by Western blot for NOS3 expression; n = 4 pairs. Grey diamond and bars, aVEC; white diamond and bars, vVEC. CoCl_2_ indicates 100 µM cobalt chloride; IFNγ, 100 ng/mL IFN-γ; rVEGF, recombinant VEGF-A; TNFα, 5 ng/mL TNF-α. Datasets A–C: Mean ± S.D. 2-Way ANOVA statistics are shown above the graph; I = interaction between treatment and valve side; T = treatment; S = valve side. * *p* < 0.05 compared to the control group (Dunnet test).

Western blot analysis revealed that TNF-α, but not IFN-γ, promoted HIF-1α stabilization only in aVEC after 24 h of treatment (Figure 3A). However, the interplay between IFN-γ and TNF-α was necessary to promote HIF-1α stabilization in vVEC (Figure 3A). Cobalt chloride (CoCl_2_), a chemical inducer of HIF-1α, was used as positive control. HIF-1α regulates the expression of several downstream genes involved in metabolism and angiogenesis, such as VEGF-A, an important regulator of endothelial phenotype. No cytokine was able to promote detectable secretion of VEGF-A in aVEC activated for 24 h (Figure 3B).

Then, we explored NOS3 protein expression. Western blot revealed that TNF-α, but not IFN-γ, triggered NOS3 protein downregulation in aVEC and vVEC (Figure 3C), suggesting differences in endothelial phenotype induced by these cytokines. Collectively, data revealed distinct effects between IFN-γ and TNF-α on HIF-1α and NOS3 expression in side-specific VEC under shear conditions.

### 2.4. Differential Effects of IFN-γ and TNF-α on VEC Migration under Multiaxial Flow

**Figure 4:**
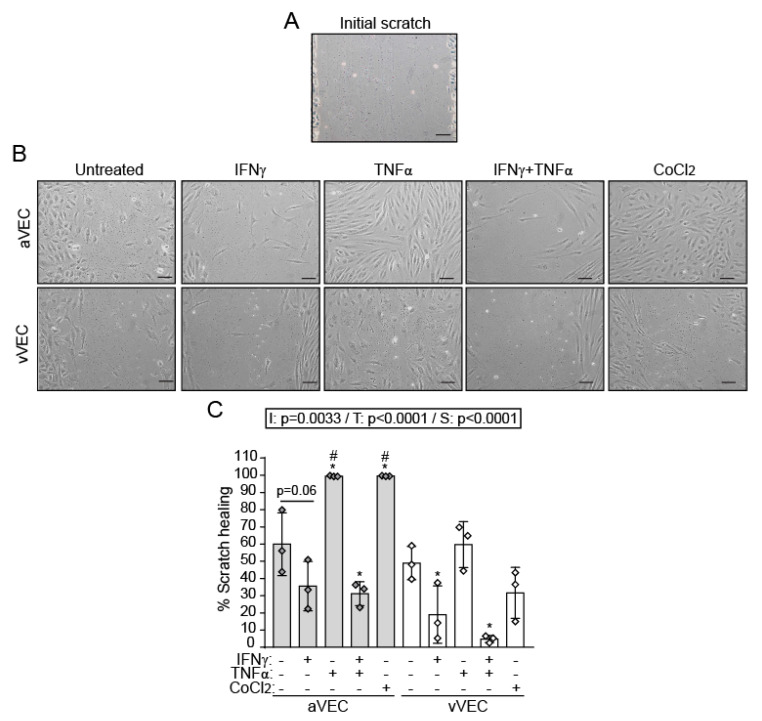
TNF-α and CoCl_2_ promote migration of aVEC under multiaxial flow conditions, while IFN-γ reduces TNF-α-mediated migration. aVEC and vVEC monolayers were sheared and activated for 24 h in parallel, and the wound-healing assay was performed as indicated in Methods. (**A**,**B**) Representative photograph of the scratch size in the aVEC and vVEC monolayers at the center of the wells (multiaxial flow), at 0 (**A**), and 24 h (**B**) time points. (**C**) % scratch healing was analyzed for each condition in n = 3 aVEC-vVEC pairs from independent valve donors. Dataset C: Mean ± S.D. 2-Way ANOVA statistics are shown above the graph; I = interaction between treatment and valve side; T = treatment; S = valve side. * *p* < 0.05 compared to the control group (Dunnet test). # *p* < 0.05 compared to the same treatment in the other group (Sidak test).

Endothelial cell migration is crucial in angiogenesis and is regulated by HIF-1α [28]. We analyzed the ability of cytokines to repair wound healing under multiaxial flow in aVEC and vVEC. Scratch assays showed that TNF-α and CoCl_2_ promoted complete wound healing in aVEC, but not in vVEC (Figure 4A–C). Conversely, IFN-γ markedly reduced wound healing in the vVEC and showed a tendency to reduce aVEC migration, and also inhibited TNF-α–mediated cell migration (Figure 4A–C). Strikingly, TNF-α–mediated responses were significantly higher in aVEC than in vVEC (Figure 4C). Collectively, the data demonstrate differences between valve side and cytokines on cell migration.

### 2.5. IFN-γ Induces Adhesion Molecule Expression and Cytokine Secretion under Shear Stress Conditions

**Figure 5:**
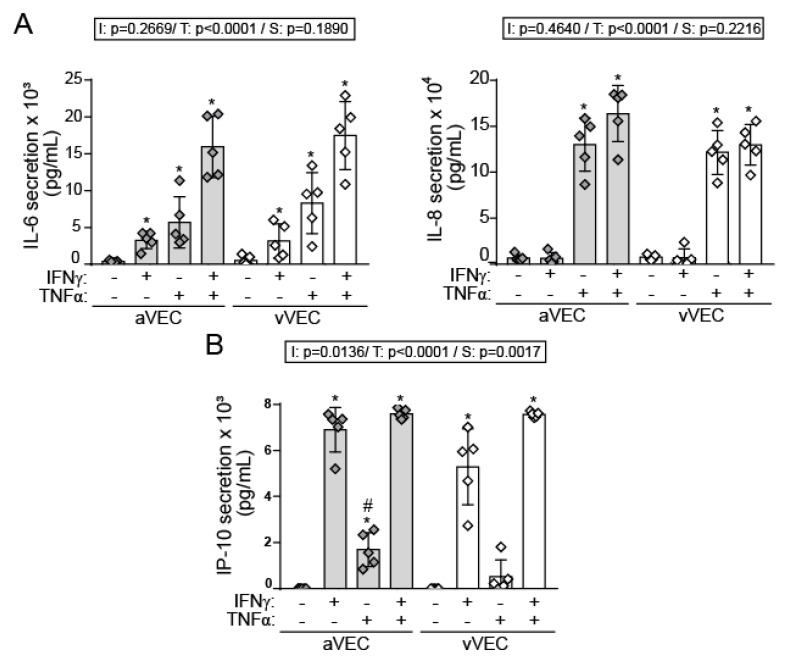
IFN-γ induces IL-6 and IP-10 secretion in side-specific VEC. (**A**,**B**) aVEC and vVEC were sheared for 48 h and then activated with the indicated ligands for 24. Supernatants were collected to assay cytokine secretion by ELISA. (**A**) IL-6 and IL-8 secretion; n = 5 aVEC and vVEC pairs. (**B**) IP-10/CXCL10 secretion; n = 5. Doses as in Figure 3. Dataset A–B: Mean ± S.D. 2-Way ANOVA statistics are shown above the graph; I = interaction between treatment and valve side; T = treatment; S = valve side. * *p* < 0.05 compared to the control group (Dunnet test). # *p* < 0.05 compared to the same treatment in the other group (Sidak test).

Next, we analyzed the inflammatory phenotype induced by IFN-γ in side-specific VEC. Enzyme linked immunosorbent assay (ELISA) analysis revealed that IFN-γ and TNF-α induced IL-6 secretion, with TNF-α being the most potent cytokine. Strikingly, both cytokines cooperated to potentiate the effect. No significant differences between aVEC and vVEC were observed (Figure 5A). In contrast, only TNF-α induced IL-8 secretion (Figure 5B), thus mirroring the gene expression profile induced by these cytokines in mixed VEC (Figure 2C). In addition, IFN-γ promoted a marked secretion of the IP-10/CXCL10, while TNF-α induced a lower secretion that was significantly higher in aVEC compared to vVEC (Figure 5B).

**Figure 6:**
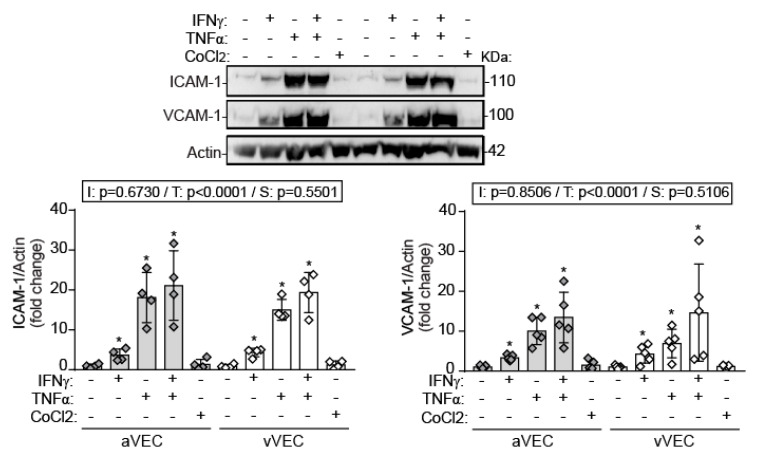
IFN-γ and TNF-α promote adhesion molecule expression in aVEC and vVEC. Cells were sheared for 48 h and treated with the indicated ligands for 24 h and whole cell extracts analyzed by Western blot for ICAM-1 and VCAM-1 expression. Densitometry analysis and representative blot of n = 4 aVEC and vVEC pairs. Doses as in Figure 3. Datasets: Mean ± S.D. 2-Way ANOVA statistics are shown above the graph; I = interaction between treatment and valve side; T = treatment; S = valve side. * *p* < 0.05 compared to the control group (Dunnet test).

Regarding adhesion molecule expression, Western blot demonstrated that IFN-γ significantly induced ICAM-1 and VCAM-1, although to a lesser extent than TNF-α, and no significant differences between aVEC and vVEC (Figure 6). To note, chemical stabilization of HIF-1α did not affect adhesion molecule expression, suggesting that this transcription factor is not involved in these inflammatory responses (Figure 6). Collectively, the data demonstrate that IFN-γ promotes a pro-inflammatory phenotype in VEC, exhibiting differences with TNF-α.

### 2.6. IFN-γ and TNF-α Increased THP-1 Adhesion to a Higher Extent in aVEC Cultured under Multiaxial Flow Conditions

**Figure 7:**
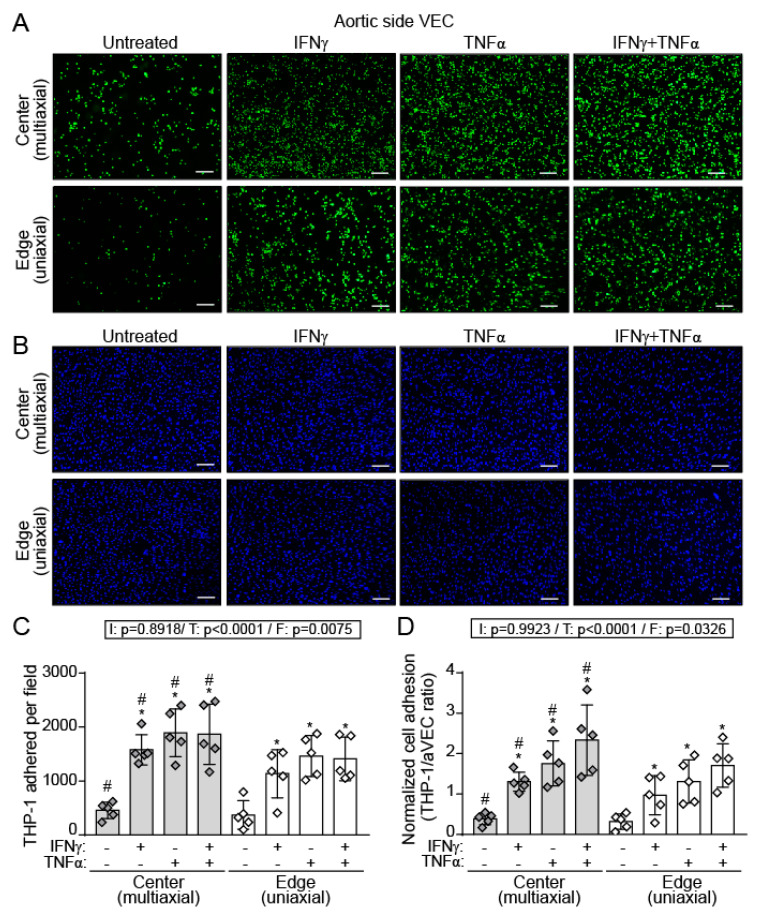
IFN-γ and TNF-α promote monocyte adhesion to aVEC monolayers to a higher extent under multiaxial flow conditions. (**A**,**B**) aVEC monolayers were sheared and adhesion assays performed as indicated in Methods. (**A**,**B**) Representative microphotographs of calcein-stained THP-1 cells adhered to aVEC (**A**) or DAPI-stained aVEC at the edge and the center of the wells (**B**); 115X magnification. (**C**) Data correspond to the number of adhered THP-1 cell per field. (**D**) Data were normalized to total VEC number. Images were analyzed with ImageJ software of n = 5 aVEC isolates for each group. White line indicates 100 µm. Doses as in Figure 3. F indicates flow pattern. Datasets C–D: Mean ± S.D. 2-Way ANOVA statistics are shown above the graph; I = interaction between treatment and flow pattern; T = treatment; F = flow pattern. * *p* < 0.05 compared to the control group (Dunnet test). # *p* < 0.05 compared to the same treatment in the other group.

Immune cell infiltration is one of the earliest events in CAVD [1,2,4,5]. To shed light into this process, we investigated the influence of multiaxial and uniaxial flow patterns on the adhesion of monocytes to the valve endothelium. Adhesion assays with THP-1 cells demonstrated that IFN-γ and TNF-α increased monocyte adhesion to aVEC cultured under both multiaxial and uniaxial flows (Figure 7A–D). Remarkably, the number of THP-1 adhered (Figure 7C) and the THP-1/VEC ratio (Figure 7D) revealed significantly higher adhesion in the center (multiaxial) compared to the edge (uniaxial) of the wells. Moreover, TNF-α was the most potent stimulus, and further cooperated with to IFN-γ to potentiate the pro-adhesive effect (Figure 7D). Of note, IFN-γ and TNF-α co-stimulation reduced the number of aVEC, although in a similar manner at the center and the edge of the wells (Supplemental Figure S2). Together, data demonstrate flow-specific effects of IFN-γ and TNF-α on monocyte adhesion to aVEC monolayers.

## 3. Discussion

**Figure 8:**
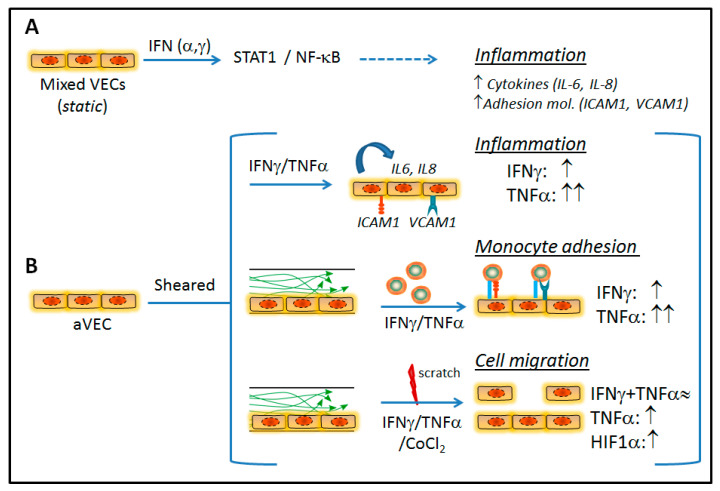
Schema depicting the effects of IFN in shear-stressed site specific VEC. (**A**) In mixed VEC under static conditions, IFN promotes a pro-inflammatory phenotype characterized by the induction of STAT1 and NF-κB routes and subsequent inflammatory molecules. (**B**) Exposure of sheared side-specific VEC to cytokines (IFN-γ, TNF-α) promotes inflammation. Moreover, aVEC were more prone to cytokine-mediated monocyte adhesion under multiaxial flow conditions. Finally, TNF-α and a chemical inducer of HIF-1α (CoCl_2_) promoted migratory effects, while IFN-γ reduced TNF-α–mediated effect. Together, data revealed differences between cytokines and flow types.

This study reports IFNs as pro-inflammatory cytokines in VEC isolated from human aortic valves. IFN-γ triggers a pro-inflammatory, pro-adhesive, and anti-migratory phenotype in sheared aVEC and vVEC. Moreover, IFN-γ exhibits major differences and interplay with TNF-α, the most studied and prototypical pro-inflammatory cytokine in VEC [10,12]. Data also disclose striking side-specific effects of cytokines on cell migration, as well as increased monocyte adhesion to aVEC sheared under multiaxial flow, thus supporting the notion of laminar flow as a protective flow pattern in valve endothelium. Altogether, these data, summarized in Figure 8, suggest that IFNs could contribute to the progression of CAVD at the early inflammatory stages.

Aortic valve endothelium may be exposed to type I and II IFN from different sources, such as the circulation or from cells residing in the valve due to infection or tissue injury. IFN-γ, the only member of the type II IFN family, has been detected in calcified aortic valves and reduces the calcium resorption potential of osteoclasts, potentially contributing to calcification in CAVD [17]. Whether type I IFN are present in valve cusps remains unexplored, but they can be secreted by VIC challenged with pathogen molecular patterns [29]. Importantly, a rare interferonopathy, the atypical Singleton—Merten Syndrome, directly links type I IFN activity with aorta and aortic valve calcification in the young [30,31].

This study highlights the pro-inflammatory and anti-migratory effects of IFNs in human endothelial valve cells. Data extend our previous findings demonstrating IFN-triggered inflammation and osteogenesis in human VIC [13,14]. IFNs and its downstream JAK/STAT routes play a critical role in the regulation of both innate and adaptive immunity [15,16]. Remarkably, dysregulated JAK/STAT signaling has been previously linked to disease. In the cardiovascular context, STAT1 activation has been associated with ischemic disease progression [32], and IFNs are known to contribute to plaque progression in atherosclerosis [27]. Importantly, these pathways are relatively safe and druggable therapeutic targets, as demonstrated by the use of JAK inhibitors in clinics for treatment of polycythaemia vera, and rheumatoid arthritis among others [33]. In this study, initial experiments under static conditions revealed that human VEC sense type I and II IFNs, which triggers the activation of STAT1 and NF-κB, and the subsequent expression of inflammatory mediators. Additional pathways, like STAT3, could also play a role in JAK/STAT signaling in valve cells, given that macrophage recruitment promotes calcification and alters STAT3 splicing in the Notch1(+/−) model of CAVD [34]. The disease occurs in a side-specific manner, likely mediated by the significant differences in hemodynamic forces experienced by each side of the aortic valve [1,4,22]. Our study in side-specific VEC focused on IFN-γ effects given its association to atherosclerosis [27], and its presence in diseased valves [17]. The effects of type I IFNs in side-specific VEC remain to be addressed in future studies.

A remarkable finding of this study is the hypoxia-independent stabilization of HIF-1α by inflammatory mediators in sheared aVEC and vVEC. In aVEC, TNF-α was sufficient to induce HIF-1α stabilization, whereas co-stimulation with IFN-γ was necessary to stabilize HIF-1α in vVEC. These findings are reminiscent of the TNF-α–mediated stabilization of HIF-1α reported in different cancer cell lines [35]. Furthermore, in the cardiovascular system, HIF-1α stabilization via NF-κB in vascular endothelial cells promoted endothelial dysfunction in atheroprone regions [36]. In the aortic valve context, the activation of HIF-1α by disturbed flow led to endothelial-to-mesenchymal transition and subsequent calcification of VEC [37]. Therefore, one may hypothesize that the immune-mediated activation of HIF-1α could be a novel mechanism accounting for the reported TNF-α–induced effects in VEC [11,12]. Here we also show that TNF-α induced *VEGFA* gene expression in mixed VECs, but TNF-α or HIF-1α stabilization did not induce detectable VEGF-A secretion by aVEC. These data are consistent with VEGF-A being produced but not secreted by endothelial cells, where it may mediate intracellular effects [38]. Further studies are needed to elucidate VEGF-A intracellular effects in VEC.

IFN-γ, in contrast to TNF-α, did not affect NOS3 expression in side-specific and sheared VEC. These findings are in line with previous reports in which IFN-γ failed to affect NOS3 activity in bovine aortic endothelial cells [39]. Remarkably, our data argue for cytokine-specific effects on valve endothelial function, given the reported protective role for endothelial-derived NO in the aortic valve context [10,40]. Previous reports in porcine VEC demonstrated that TNF-α triggers NOS3 downregulation [12]. Our study confirms these findings in human cells. NOS3 expression has been reported to be higher in the aortic side in porcine VEC exposed to 20 dynes/cm2 steady shear [41]. However, we did not find differences in basal NOS3 expression or upon stimulation comparing aVEC to vVEC. This discrepancy may be explained by different species and/or flow settings.

Wound healing assays revealed side- and cytokine-specific effects on cell migration. Indeed, IFN-γ inhibited cell migration in vVEC and showed a tendency to reduce aVEC migration, which correlates with previous studies demonstrating the anti-angiogenic and anti-migratory effects of this cytokine [42]. In contrast, TNF-α induced wound closure only in aVEC monolayers after 24 h of treatment. Interestingly, IFN-γ showed a more prominent role in regulating cell migration, given that it was able to inhibit TNF-α–induced cell migration. One might speculate that IFN-γ could reduce VEC migration, thus affecting the maintenance and repair of endothelium after injury. Additionally, CoCl_2_, a chemical inducer of HIF-1α, promotes aVEC migration, indicating a role of HIF-1α in regulating this process. This is consistent with the reported crucial role of HIF-1α during the migration of human osteosarcoma cells [43]. Our data also suggest the involvement of additional pathways, given that the IFN-γ–mediated anti-migratory effects seem to outweigh the HIF-1α effects of TNF-α. Further studies to elucidate the mechanism are warranted. Consistent with our data, IFN-α and STAT1 arrest monocyte migration by modulating RAC/CDC42 pathways, whereas STAT3 enhances migration in monocytes [44]. In addition, several reports associate IFN-γ or JAK/STAT with increases in cell migration in the cancer context [45,46]. Altogether, this evidence indicates that the JAK/STAT pathways regulate cell migration in a context-dependent manner.

IFN-γ triggered a pro-inflammatory phenotype in side-specific VEC, characterized by the induction of adhesion molecule expression, and secretion of the IFN-related chemokine IP-10/CXCL10, and IL-6. Moreover, IFN-γ cooperated with TNF-α to enhance IL-6/IL-8 secretion. This may be of relevance since IL-6 is a cytokine associated with calcification and CAVD pathogenesis [5]. The lack of effect on IL-8 secretion by IFN-γ may suggest a cytokine-specificity for IFN-γ similar to that reported for human microvascular endothelial cells [47]. Remarkably, we found increased TNF-α–mediated secretion of IP-10/CXCL10 in aVEC compared to vVEC. These data could be physiologically relevant given that IP-10 is a potent chemoattractant for monocytes and T cells that favors atherosclerosis development in mice models by regulating the local balance of immune cells [48].

The aortic endothelial layer is exposed to oscillatory and low-shear flow, which has been linked to atheroprone regions in their vascular counterparts [23]. Differences between aortic and ventricular VEC have been reported on the regulation of aortic valve calcification, NO expression, mechanical properties, and transcriptional profile, with aVEC expressing lower levels of protective genes [21,24,25]. However, the potential effect of oscillatory flow on immune cell adhesion to aVEC remains unexplored. Our data suggest a potential interplay between inflammatory cytokines and oscillatory flow on monocyte adhesion that may be relevant to CAVD. In fact, infiltrated monocyte-derived cells, such as macrophages, are enriched in calcified valves and show increased inflammatory and osteogenic activity [34,49,50]. Moreover, the enrichment of polarized macrophages in bicuspid valves and the activation of macrophages by cyclic mechanical strain in several models suggest a potential role for mechanical strain in macrophage activation and the ensuing inflammation in CAVD [49]. Here we provide evidence indicating that aortic-sided endothelium is more prone to IFN-γ–mediated monocyte adhesion under multiaxial flow as compared to uniaxial flow, and report cooperation with TNF-α. Oscillatory flow may alter VEC phenotype and potentiate the response to cytokines. In fact, the existence of mechanosensors and shear-sensitive genes leading to alterations in cellular phenotype and inducing inflammation and valve dysfunction have been reported in aortic valve endothelium [51]. Even though the shear stress in our system does not reproduce the physiological conditions, data suggest that the oscillatory flow experienced by the aortic surface of the valve may favor the adhesion of circulating immune cells after endothelial damage or inflammation. Furthermore, this finding supports the notion of laminar flow as a protective pattern in endothelial cells [23,52]. Comparison between cytokines revealed TNF-α as a stronger inducer than IFN-γ on adhesion molecule expression and monocyte adhesion. To note, in vascular endothelial cell monolayers flow had an impact on the number of cells per mm2 that increased with radial distance under all conditions [53]. In contrast, flow had no significant effect on VEC number, thus reinforcing the notion of functional differences previously reported between valvular and vascular endothelial cells [18,41,54].

The JAK/STAT pathways are well-known regulators of adhesion molecule expression in different cellular types. Indeed, Tyr and Ser phosphorylation of STAT1 is critical for ICAM-1 expression, and the STAT1/IRF1 axis activates ICAM-1 in endothelial cells from liver [55,56]. Moreover, other JAK/STAT family members, such as JAK2/STAT3 pathway, mediated adhesion molecule expression in a rat model of severe acute pancreatitis-associated lung injury and in endothelial cells [57,58]. Our data point to the involvement of NF-κB, known to play a role in the IFN response [59] and its regulated genes, as the downstream mechanism in monocyte adhesion and cytokine secretion. In fact, data from mixed VEC revealed early activation of STAT and a later activation of NF-kB upon IFN exposure, and subsequent induction of adhesion molecule expression, namely ICAM-1 and VCAM-1, which are transcriptionally regulated by NF-κB in endothelial cells [60].

In the valvular context, we and others have shown that NF-κB inhibition decreases ICAM-1 expression induced by TLR4 activation in aortic valve interstitial cells and the correlation of greater production of ICAM-1 in response to TLR4 stimulation with enhanced NF-κB activation in AVIC of stenotic valves [29,61], evidence supporting a major role for NF-κB in the regulation of adhesion molecule expression in VEC. In addition to NF-κB, these adhesion molecules are also regulated by other transcription factors. For example, PI3K and MAPK/AP-1 routes play key roles on regulating ICAM-1 and VCAM-1 expression in monocytes and endothelial cells [62,63]. Exploring these pathways and their potential contribution to the effects of IFNs in VEC will be part of further studies.

There are several limitations to the experimental model used in this study. Firstly, the swirling system used in the study simulates different flow patterns, but the shear stress values differ from those experienced by cells under physiological conditions, although it is still a valid method since, between the edge and the center, there is a difference in the amount of shear stress [64], and a similar relationship exists between the aortic and ventricular VECs [22]. In addition, VEC also experience other mechanical forces, namely cyclical stretch, although it should be noted that these two forces are hard to examine simultaneously. Moreover, Ghim and colleagues developed a method for segmenting VEC growth in the edge or the center of the wells [53], therefore allowing the study of the effects of each flow pattern individually. In our conditions, the behavior of cells from each location could have been influenced by cells from the other locations and could have concealed some side-specific responses evaluated in cell supernatants, i.e., cytokine secretion. The implementation of that culture system would be of interest to explore the individual influence of different flow patterns beyond cell adhesion experiments. Additionally, the scratch assay may have some limitations due to the putative contribution of cell proliferation, although it was a useful tool to disclose side- and cytokine-specific differences in cell migration.

Collectively, our data unveiled new findings on the inflammatory effects of IFNs in human VEC, showing differences with TNF-α on migration and NOS3 downregulation. We also describe that multiaxial flow conditions may increase the adhesion of immune cells to inflamed valve endothelium. Overall, this study provides new findings that could be relevant to understand the initial inflammatory stages of the disease and support the study of JAK/STAT pathways as potentially relevant routes in CAVD.

## 4. Materials and Methods

### 4.1. Endothelial Valve Cell Isolation

VEC, isolated simultaneously from both sides of the valve (mixed VEC), were obtained from tricuspid aortic valves of cardiac transplant recipients who had no history of valve disease and a multi-organ donor at the Hospital Clinico Universitario de Valladolid (Supplemental Table S1, see Supplementary Materials). Side-specific VEC (aVEC and vVEC) were isolated from valves obtained from the Royal Brompton Heart Valve Bank. The valves used were classed as unsuitable for clinical application (Supplemental Table S2). A mixed VEC population was cultured as previously described [14,65]. Briefly, valve cusps were digested with a 2.5 mg/mL of type II collagenase solution (Gibco Invitrogen, Amarillo, TX, USA; #17101–015) for 15 min at 37 °C. Then, VEC were isolated by centrifugation, plated onto culture flasks, and cultured as indicated below. Elimination of contamination of interstitial cells was performed by cell sorting, as described [14], and flow cytometry post analysis revealed that the percentage of PECAM/CD31+ cells ranged from 95–98%. For side-specific VEC isolation, a custom-made device allowing single-side exposition to a collagenase solution was used, as previously described [66]. Cell phenotype was confirmed by the expression of endothelial markers, platelet-endothelial cell adhesion molecules (PECAM/CD31) and Von Willebrand factor (VWF) (Supplemental Figure S1).

### 4.2. Cell Culture

Endothelial cells were cultured in Endothelial Cell Growth Medium (EGM-2; Lonza, Basel, Switzerland; #C22011) supplemented with 10% heat inactivated fetal bovine serum (FBSi), antibiotic-antimycotic solution (100 U/mL penicillin and 100 μg/mL streptomycin; Gibco Invitrogen, USA; #15240-062), 1% L-glutamine (Lonza, Switzerland; #BE17-605) and 4% EGM-2 supplement (Lonza, Switzerland; #C39216). Cells were grown in six-well plates pre-coated with 1% gelatin (Sigma-Aldrich, St. Louis, MO, USA, #G1393) in phosphate saline buffer (PBS) for 30 min at 37 °C.

The human monocytic cell line THP-1 was obtained from the American Type Culture Collection (US). Cells were cultured in RPMI 1640 (BioWhittaker, USA; #BW09-774E) supplemented with 10% FBSi, 2 mM L-glutamine, and antibiotic-antimycotic solution. All cellular types were maintained in an incubator containing humidified air with 5% CO_2_ at 37 °C.

### 4.3. Cell Activation under Static and Shear Stress Conditions

Cells were serum-starved overnight in EGM-2 supplemented with 2% FBSi, and then activated with the indicated stimulus. Mixed VEC monolayers were activated under static conditions. Monolayers of side-specific VEC were sheared using an orbital shaker swirling system that creates different patterns of shear, a multidirectional flow pattern in the center of the wells and a high magnitude uniaxial flow in the edge of the wells, a method previously used for vascular endothelial cells [64]. Briefly, 150,000 side-specific VEC were seeded in six-well plates pre-coated with 1% gelatin and cultured overnight. The next day, medium was replaced by 1.9 mL of EGM-2 and plates were placed on an orbital shaker platform of 5 mm radius and angular velocity of 150 rpm. After 48 h of exposure to flow, cells were activated without changing medium and cultured for additional 24 h.

Recombinant cytokines included IFN-α (100 ng/mL, specific activity ≥ 1.8 × 108 IU/mg; Peprotech, London, UK; #500-P32AG), IFN-γ (100 ng/mL, specific activity ≥ 2 × 107 IU/mg; Peprotech, UK; #300-02) and TNF-α (5 ng/mL; Cell Biolabs, San Diego, CA, USA; #CO 12105). Cobalt chloride (CoCl_2_; # C-8661 Sigma Aldrich, St. Louis, MO, USA) was used as a chemical stabilizer of HIF-1α.

### 4.4. Protein Analysis by Immunoblot

VEC activated under static conditions were lysed with TNE buffer (Tris-HCl 20 mM pH 7,4, NaCl 150 mM, EDTA 5 mM, NP-40 0.01%, supplemented with protease and phosphatase inhibitors) as in previous reports [29]. Side-specific VEC monolayers, sheared and activated as indicated above, were lysed in RIPA buffer supplemented with protease inhibitor cocktail as in previous studies [24]. The bicinchoninic acid assay (BCA, Thermo Scientific, Waltham, MA, USA; #23227) was used to measure total protein content. Cell lysates containing 25 μg of protein were separated by SDS-PAGE and proteins were transferred onto membranes, PVDF (for VEC) or nitrocellulose (for side-specific VEC). Blots were incubated overnight at 4 °C with the following primary antibodies: rabbit anti-human ICAM-1 (Santa Cruz Biotech, Dallas, TX, USA, #sc-7891), endothelial NO synthase (NOS3) (BD Biosciences, San Jose, CA, USA; #610299), pSTAT1-Tyr701 (Cell Signaling, Danvers, MA, USA; #9167), STAT1 (Cell Signaling, USA; #9172), and phosphorylated nuclear factor κ-light-chain-enhancer of activated B cells (NF-κB)-p65-Ser536 (Cell Signaling, USA; #3033), mouse anti-human HIF-1α (BD Biosciences, USA; #610958), VCAM-1 (Santa Cruz Biotech, USA; #sc-13160), β-Tubulin (Sigma-Aldrich, USA; #T7816), or goat anti-human actin (Santa Cruz Biotech, USA; #sc-1615). Horseradish peroxidase-conjugated secondary antibodies included goat anti-rabbit IgG (Agilent Technologies, Santa Clara, CA, USA, #P0448), goat-anti-mouse IgG (Bio-Rad, Hercules, CA, USA; #170-6516) (Agilent Technologies, USA; #P0447) and rabbit anti-goat IgG (Agilent Technologies, USA; #P0449). Membranes were incubated with HRP blotting substrate (ECL; Thermo Scientific, USA, #32106), imaged on X-ray films, and bands were analyzed by QuantityOne Software (Bio-Rad, USA). Data were expressed as arbitrary units normalized to β-tubulin or actin and to basal conditions. Original blots are enclosed in the Supplementary File.

### 4.5. Reverse Transcriptase Quantitative PCR (RT-qPCR)

VEC monolayers were activated for 24 h and total RNA was isolated using TRI Reagent (Invitrogen, Carlsbad, CA, USA; #AM9738) following manufacturer’s protocol. Next, 1.5 µg of first strand cDNA were generated by M-MLV reverse transcriptase (Invitrogen, Carlsbad, CA, USA; #28025-013), and 20 ng were later amplified by PCR using SYBRgreen master mix (Kapa Biosystems, Wilmington, MA, USA; #KK4610) with the corresponding primers.

Glyceraldehyde 3-phosphate dehydrogenase (GAPDH):

Forward 5′-TGCCAAATATGATGACATCAAGAA-3′;

reverse 5′-GGAGTGGGTGTCGCTGTTG-3′.

IL6: Forward 5′-CACCTCTTCAGAACGAATTG-3′;

reverse 5′-CTAGGTATACCTCAAACTCC-3′.

IL8: Forward 5′-ATGACTTCCAAGCTGGCCGT-3′;

reverse 5′-TCCTTGGCAAAACTGCACCT-3′.

NOS3: Forward 5′-CCAGCTAGCCAAAGTCACCAT-3′;

reverse 5′-GTCTCGGAGCCATACAGGATT-3′.

VEGFA: Forward 5′-ATCTGCATGGTGATGTTGGA-3′;

reverse 5′-GGGCAGAATCATCACGAAGT-3′.

qPCR reaction was carried out using a LightCycler480^®^ (Roche Diagnostics, Rotkreuz, Switzerland). Two technical replicates were performed, and the 2^−ΔΔCt^ method was used for calculations.

### 4.6. Cell Migration Assay

The migratory potential of side-specific VEC was analyzed at the center of the wells (multiaxial flow) using the wound healing or scratch assay [67]. First, cells were seeded in 6-well plates for 36 h and serum-starved overnight in EGM-2 supplemented with 2% FBSi. The next day a scratch was created using a P200 pipette tip from the top to the bottom of each well, and dead cells were removed washing twice with pre-warmed medium. Microphotographs of the scratch area were taken under a bright field microscope (time 0). VEC were then activated with the indicated stimuli in 1.9 mL of fresh EGM-2 supplemented with 2% FBSi and sheared for additional 24 h. Then, cells were washed twice with medium and five images of the scratch space were taken at 24 h time point. Cell migration in the recovery space was estimated by using ImageJ software (NIH, Bethesda, MD, US), and data was expressed as % of scratch healing calculated as follows:

$$\%\;\mathrm{wound}\;\mathrm{healing}=\frac{\mathrm{Original}\;\mathrm{area}\;\left(0\;h\right)-\mathrm{Treatment}\;\mathrm{area}\;\left(24\;h\right)}{\mathrm{Original}\;\mathrm{area}\;\left(0\;h\right)}\times 100$$

### 4.7. Cytokine Secretion by ELISA

Side-specific VEC monolayers were sheared and activated as indicated. Cytokine secretion was assessed in the supernatants by ELISA. Secretion of IL-6 (Diaclone, Besançon, France; #950.030.048), IL-8 (Diaclone, France; #950.050.096), and IFN-γ–induced protein 10 (IP-10) (Diaclone, France; #850.950.096) was evaluated by immunoassay kits following manufacturer’s protocol. Absorbance at 450 nm was measured using a microplate reader, Versamax (Molecular Devices, Sunnyvale, CA, USA).

### 4.8. Monocyte-VEC Monolayer Adhesion Assay

Side-specific VEC monolayers, sheared and activated as previously indicated, were co-cultured with THP-1 monocytic cells to analyze cell adhesion. First, 106 THP-1 cells/mL were stained with 1 µg/mL calcein-AM (Thermo Scientific, USA; #3099) for 30 min at 37 °C. After three washes, 106 THP-1 cells were resuspended in 1.5 mL and co-cultured with VEC monolayers for 1 h at 37 °C without swirling to allow adhesion. Next, cells were washed three times with medium and fixed with 4% p-formaldehyde for 10 min. After washing twice with PBS, 10 pictures of the edge and of the center of the wells were captured using a fluorescence Zeiss axioscope microscope (Carl Zeiss, Oberkochen, Germany) at 115X magnification. For normalization, cell counting of VEC monolayers sheared and activated was performed in parallel by DAPI (4’,6-diamidino-2-phenylindole) staining for 10 min (1 µg/mL; Sigma-Aldrich, USA; #D9564). Counting of calcein-stained THP-1 and DAPI-stained VEC was performed using a custom-made script in ImageJ (NIH, Bethesda, Rockville, MD, USA). Data were expressed as total number of THP-1 cells adhered per field, and normalized cell adhesion (THP-1/VEC ratio).

### 4.9. Statistical Analysis

Data are expressed as mean ± standard deviation (SD) and represented as bar chart overlaid on a dot plot, where each dot corresponds to a cell isolate from an independent valve donor. One-way or two-way ANOVA analyses followed by Dunnet post-hoc test (vs. control) were performed for data following a normal distribution and homoscedasticity requirements. For non-normal distributed or unequal variances data, a Box-Cox transformation was previously applied. Two-way ANOVA statistics are presented in a box above the graphs (treatment/side or treatment/flow). When the interaction between 2 factors (treatment/side) was significant, comparisons between cells using a Sidak test were also performed (Figure 4C and Figure 5B). *p* indicates the *p*-adjusted value, and statistical significance was considered for *p* value < 0.05. Analyses were performed using GraphPad Prism 6 (GraphPad Software Inc., SanDiego, CA, USA).

## Data Availability

The data supporting the findings of this study are available on request from the corresponding author.

## Supplementary Materials

The following are available online at https://www.mdpi.com/article/10.3390/ijms221910605/s1.

## Abbreviations

aVEC:	aortic-side valvular endothelial cells
CAVD:	calcific aortic valve disease
DAPI:	4′,6-diamidino-2-phenylindole
EGM-2:	endothelial growth medium-2
ELISA:	enzyme linked immunosorbent assay
NO:	nitric oxide
NOS3:	endothelial nitric oxide synthase
FBSi:	inactivated fetal bovine serum
HIF-1α:	hypoxia inducible factor 1α
ICAM-1:	intercellular cell adhesion molecule 1
IFNs:	interferons
IL:	interleukin
IP-10/CXCL10:	interferon gamma-induced protein 10
NF-κB:	nuclear factor κ-light-chain-enhancer of activated B cells
qPCR:	quantitative polymerase chain reaction
STAT:	signal transducers and activators of transcription
TNF-α:	tumor necrosis factor-α
VCAM-1:	vascular cell adhesion molecule 1
VEC:	valvular endothelial cells
VEGF-A:	vascular endothelial growth factor A
vVEC:	ventricular-side aortic valve endothelial cells
VIC:	valvular interstitial cells
