# Supplementary material for: Interferons Are Pro-Inflammatory Cytokines in Sheared-Stressed Human Aortic Valve Endothelial Cells
Source: Int J Mol Sci. 2021 Sep 30;22(19):10605. doi: 10.3390/ijms221910605 (PMC8508640; doi:10.3390/ijms221910605)

SUPPLEMENTAL MATERIAL

Interferons are pro-inflammatory cytokines in shear-stressed human aortic valve endothelial cells

Iván Parra-Izquierdo [1, ‡], Tania Sánchez-Bayuela[1], Javier López [2, 3], Cristina Gómez [1], J. Enrique Pérez-Riesgo [1], Alberto San Román [2,3], Mariano Sánchez Crespo [1], Magdi Yacoub [4,5], Adrian H Chester [4,5, #]*, Carmen García-Rodríguez [1,3#]*

[1] Instituto de Biología y Genética Molecular, CSIC-Universidad de Valladolid; Valladolid, Spain

[2] ICICOR, Hospital Clínico Universitario, Valladolid, Spain

[3] CIBER de Enfermedades Cardiovasculares (CIBERCV)

[4] National Heart & Lung Institute, Imperial College London, London, United Kingdom.

[5] Heart Science Centre, Magdi Yacoub Institute, Harefield, United Kingdom.

‡ Current address: Knight Cardiovascular Institute and Department of Biomedical Engineering, School of Medicine, Oregon Health & Science University, Portland, OR, USA.

METHODS

VEC characterization by immunostaining of endothelial cell markers

VEC were seeded on 1% gelatin-coated coverslips. The following day the cells were washed twice in PBS and fixed with 4% formaldehyde solution for 10 min at room temperature. Cells were then washed 3 times with PBS and permeabilized with Triton-x-100 (0.5% v/v in PBS) for 3 min and blocked for 30 min with 3% BSA in PBS. Next, immunostaining was performed by incubating cells with primary antibodies against endothelial cell markers, mouse anti-human CD31 (clone WM59; eBioscience, Waltham, MA; 1:200 in PBS-1% BSA) and a rabbit anti-human Von Willebrand factor (VWF) (Agilent Technologies, Santa Clara, CA; 1:500 in PBS-1% BSA) for 1 h at room temperature. Then, cells were incubated with secondary antibodies, Alexa Fluor 488 goat anti-mouse and Alexa Fluor 594 goat anti-rabbit IgG (Invitrogen, Carlsbad, MA; 1:1000 in PBS-1% BSA) for 1 h in the dark. Before mounting the coverslips, cell nuclei were stained with DAPI (4', 6-diamidino-2-phenylindole; 1:1000 dilution in PBS-1% BSA) for 10 min at room temperature. Permafluor aqueous mounting fluid was used as the mounting solution and the fluorescence was analysed by Ziess LSM 510 confocal microscope.

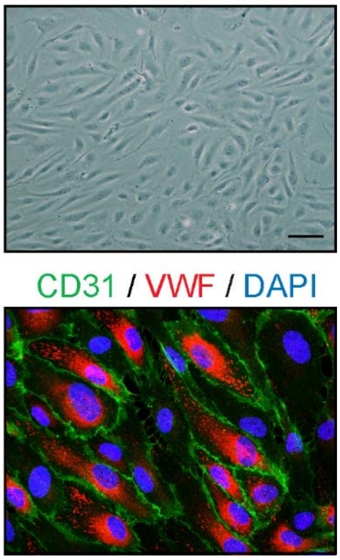

Supplemental Fig. S1. **Characterization of aortic valve endothelial cells**. Upper image, bright field microphotographs of VEC. Black line indicates 50 µm. Lower image, merged immunofluorescence images for endothelial markers of VEC (CD31 and VWF) and DAPI nuclear staining.

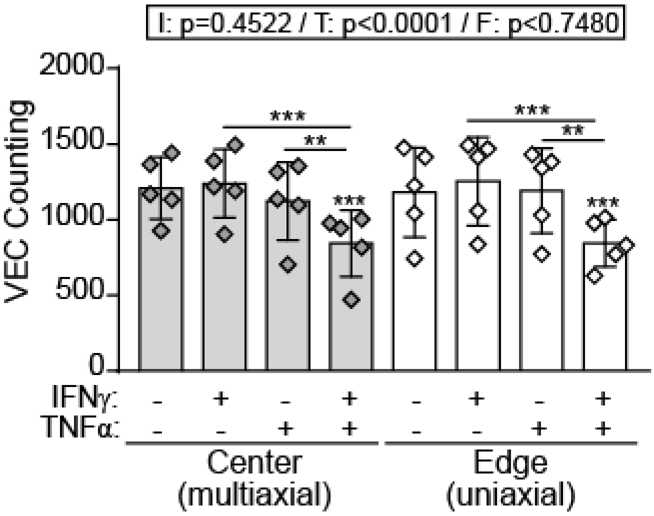

Supplemental Fig. S2. Different flow patterns do not alter aVEC counting. aVEC monolayers were sheared and activated and DAPI staining performed as indicated in Methods. Data, corresponding to Figure 7, are expressed as total aVEC number per field. N = 5 independent aVEC isolates.

SUPPLEMENTAL TABLES

Supplemental Table S1. Clinical features of patients used in the study of mixed VEC population. Data are expressed as mean average ± SEM.

**Patient characteristics (n=8)**	
Age (yr.)<br>(range)	60 ± 5<br>53-66
Sex	8 male
Etiology of heart failure	2 idiopathic<br>5 ischemic

Supplemental Table S2. Clinical characteristics of patients used in the study of aortic- and ventricular-sided endothelial cells. Data are expressed as mean average ± SEM.

**Patient characteristics (n=10)**	
Age (yr.)<br>(range)	51 ± 7<br>20-65
Sex	5 male, 5 female
Etiology of heart failure / cause of death	3 intracranial hemorrhage<br>1 congenital heart disease<br>1 hypoxic brain injury<br>3 dilated cardiomyopathy<br>1 cancer<br>1 ischemic heart disease

ORIGINAL BLOTS

Interferons are pro-inflammatory cytokines in shear-stressed human aortic valve endothelial cells, by Parra-Izquierdo et al

FIGURE 1A left

FIG 1A-left: pSTAT1

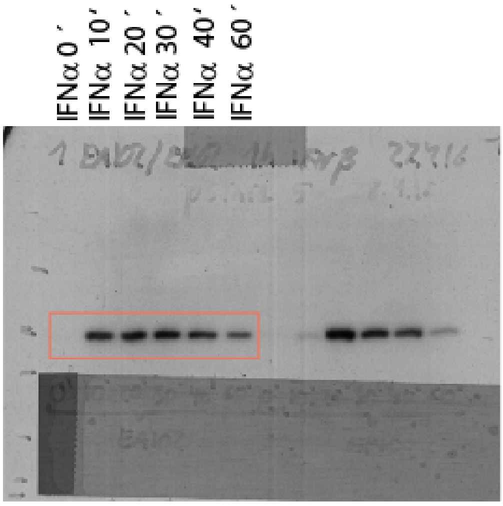

FIG 1A-left: STAT1

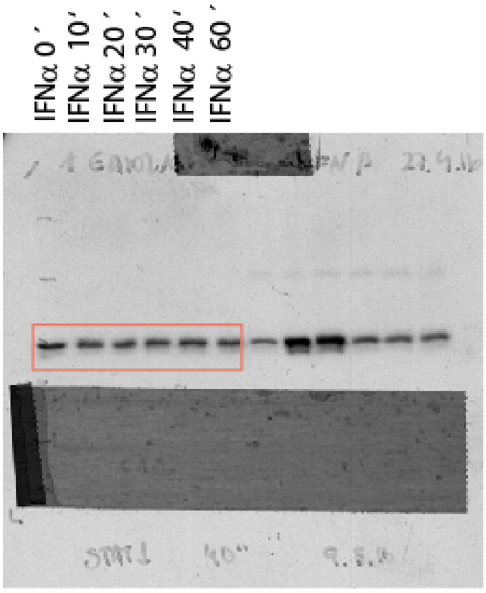

FIG 1A-left: β–Tubulin

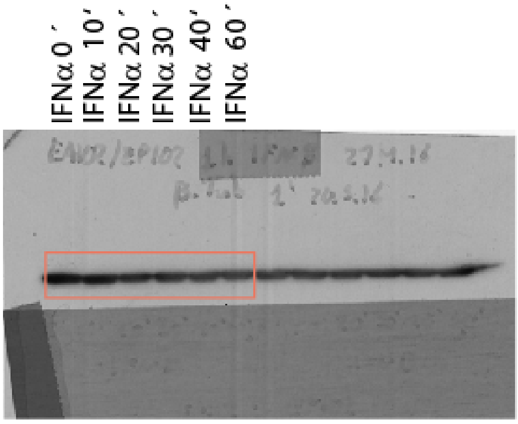

FIGURE 1A right

FIG 1A-right: pSTAT1

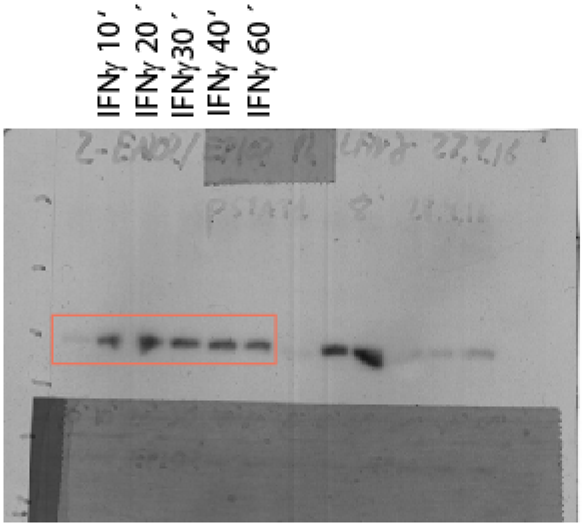

FIG 1A-right: STAT1

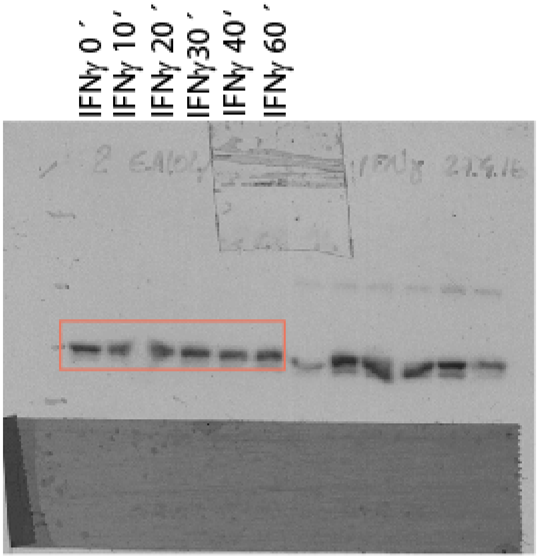

FIG 1A-right: β–Tubulin

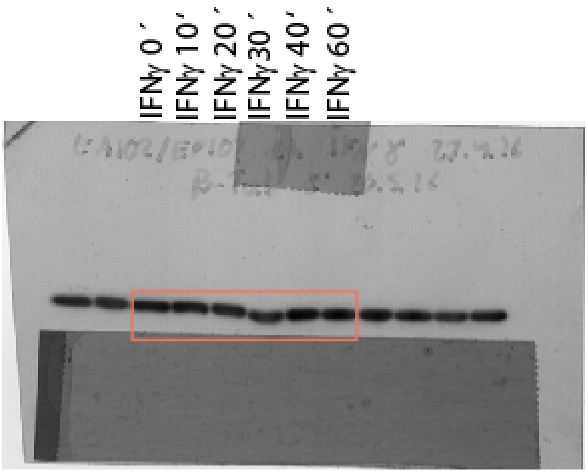

FIGURE 2A

FIG 2A: pNF-κB

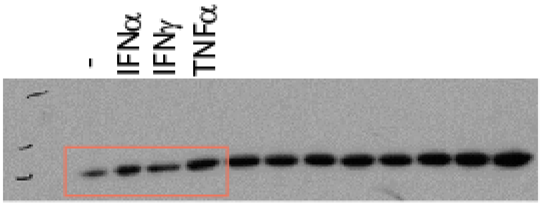

FIG2A: β-Tubulin

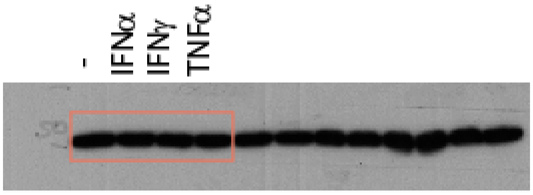

FIGURE 2B

FIG 2B-left: ICAM1

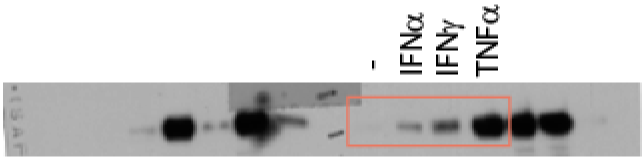

FIG2B-left: β-Tubulin

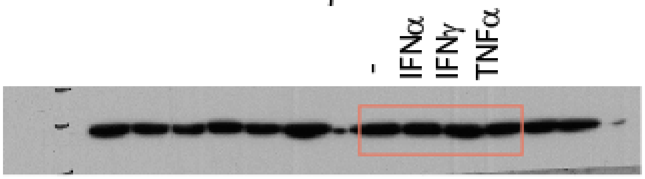

FIG 2B-right: VCAM1

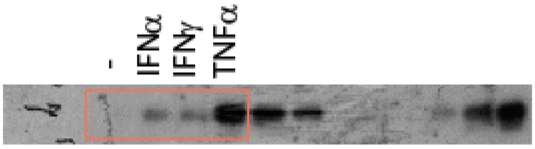

FIG2B-right: β-Tubulin

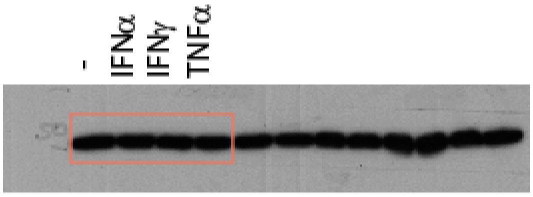

FIGURE 3A

FIG 3A: HIF1α

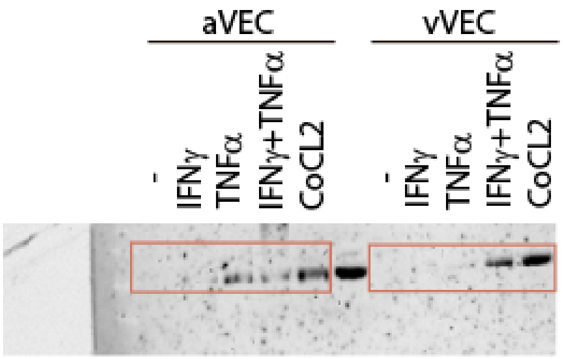

FIG3A: Actin

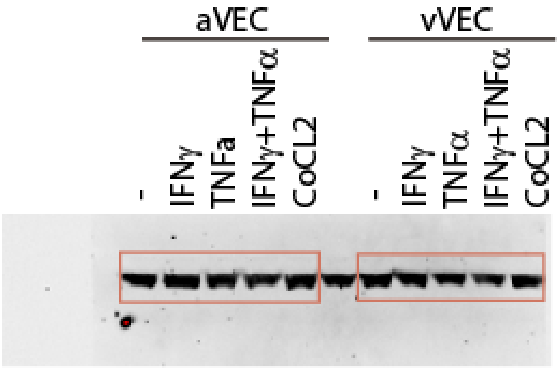

FIGURE 3C

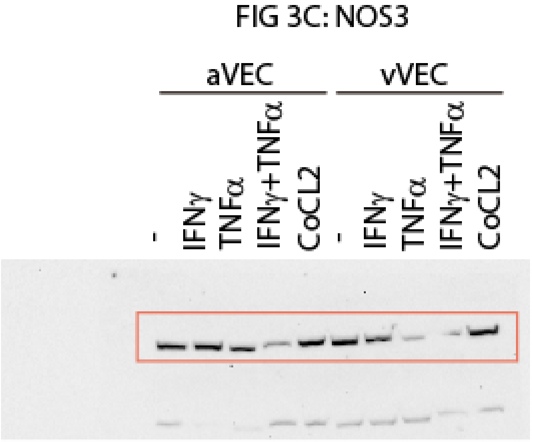
FIG 3C: NOS3
aVEC
vVEC
-
IFNγ
TNFα
IFNγ+TNFα
CoCL2
-
IFNγ
TNFα
IFNγ+TNFα
CoCL2

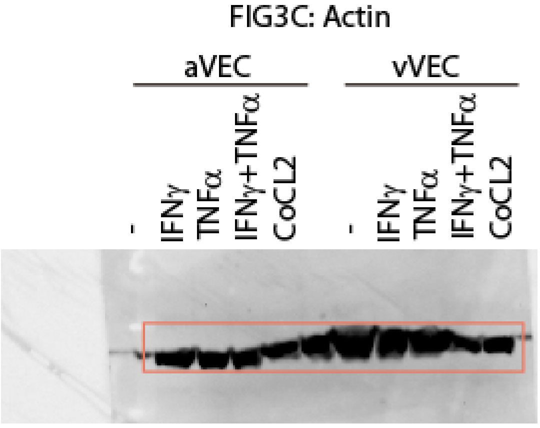
FIG3C: Actin
aVEC
vVEC
-
IFNγ
TNFα
IFNγ+TNFα
CoCL2
-
IFNγ
TNFα
IFNγ+TNFα
CoCL2

FIGURE 6

FIG 6: ICAM1

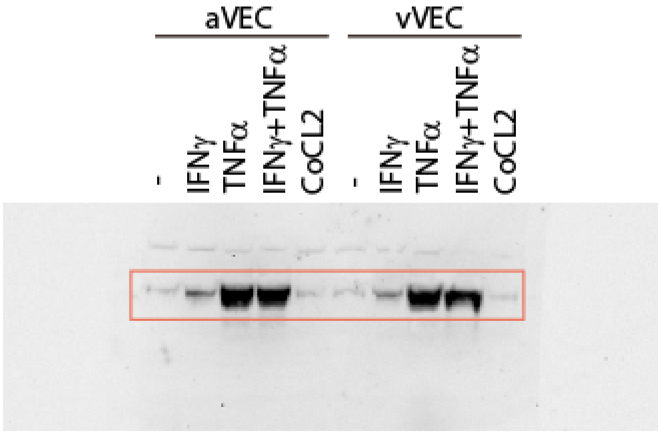

FIG6: VCAM1

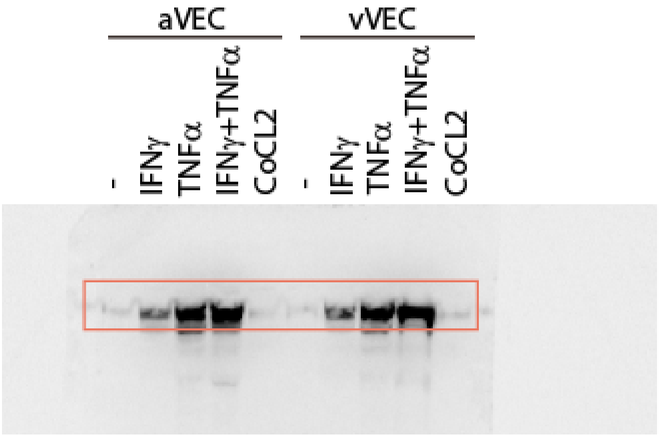

FIG6: Actin

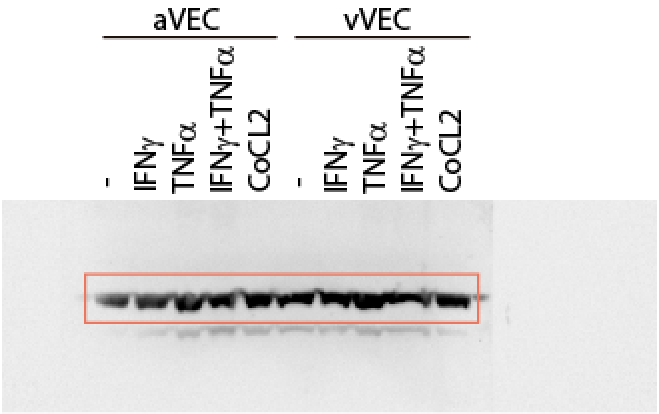

Supplement: Supplementary file 1 [file ijms-22-10605-s001.zip › ijms-1346443-supplementary.pdf]
